# Polar Fourier transform in practice: its efficiency and characteristics in reconstructing radially acquired MRI images

**DOI:** 10.1007/s10334-025-01284-w

**Published:** 2025-08-12

**Authors:** Fatemeh Rastegar Jooybari, Ali Aghaeifar, Elham Mohammadi, Klaus Scheffler, Abbas Nasiraei-Moghaddam

**Affiliations:** 1 Department of Biomedical Engineering, Amirkabir University of Technology (Tehran Polytechnic), 424, Hafez Ave., Tehran, Iran; 2 Department of Medical Biophysics, University of Toronto, Toronto, ON Canada; 3 Max Planck Institute for Biological Cybernetics, Tübingen, Germany; 4 Department for Biomedical Magnetic Resonance, University of Tübingen, Tübingen, Germany; 5 Department of Aerospace & Mechanical Engineering, University of Southern California, Los Angeles, USA; 6 Faculty of Electrical Engineering, Shahid Beheshti University, Tehran, Iran

**Keywords:** Non-Cartesian MRI, Radial acquisition, Image reconstruction, Integrated reconstruction, Fast imaging

## Abstract

**Objective:**

The Polar Fourier Transform (PFT) has been proposed as a direct alternative to gridding for reconstructing radially acquired MRI data. This study evaluates the feasibility of inline PFT implementation on a clinical MRI scanner and assesses its computational performance and image quality under acceleration.

**Materials and methods:**

PFT was implemented as modular components within the Siemens Image Calculation Environment, using a recursive numerical Hankel transform. Phantom and in vivo brain datasets acquired with 2D radial trajectories were reconstructed using both PFT and vendor-supplied gridding. Reconstruction time, SNR, artifact behavior, and spatial resolution were assessed across multiple undersampling levels (up to 8 ×), using simulations and repeated scans.

**Results:**

PFT was successfully integrated with a runtime of ~ 6–9 × acquisition time. It exhibited spatially variant behavior, concentrating resolution in central region while shifting undersampling-induced blurring outward. Compared to gridding, PFT reduced structured streaks and better preserved image quality under acceleration. Gradient delay artifacts were reduced by alternating spoke polarity. Notably, the pituitary gland and basilar artery remained visible at high acceleration, highlighting preserved central fidelity.

**Discussion:**

PFT enables effective inline reconstruction for radial MRI and preserves image quality in small central regions of interest under aggressive undersampling—supporting dynamic and ROI-focused applications.

## Introduction

Magnetic resonance imaging (MRI) captures data in the spatial frequency domain, known as k-space, and reconstructs images using the Fourier Transform (FT). In principle, k-space can be sampled along arbitrary trajectories [1, 2]. The first MR imaging experiment, introduced by Lauterbur [3], employed a radial k-space acquisition. However, MRI became clinically viable only after the development of the “spin warp” technique—also known as phase encoding—which enabled k-space sampling along Cartesian trajectories [4]. Cartesian sampling is inherently compatible with the fast Fourier Transform (FFT), the most efficient algorithm for computing FTs [5], which requires uniformly spaced data points. Thus, phase encoding in Cartesian coordinates became established as the acquisition counterpart to FFT-based reconstruction, and Cartesian sampling has since become the predominant k-space trajectory in clinical MRI.

Radial sampling remains a valuable approach in several MRI applications, owing to its intrinsic advantages [6–11]. Notably, all radial spokes pass through the center of k-space, which improves motion robustness and facilitates correction of motion-related artifacts [8]. Another important advantage is its inherent variable-density sampling, where k-space is sampled most densely at the center—a characteristic previously proposed to suppress low-frequency aliasing artifacts and reduce their perceptual impact under undersampling conditions [12].

In the absence of a fast and direct Fourier Transform algorithm applicable to polar coordinates, radially acquired k-space has traditionally been resampled onto a Cartesian grid using a process known as gridding. Although gridding is computationally efficient, it can introduce artifacts or degrade image quality—especially under high levels of undersampling, as used in accelerated imaging [10]. Additionally, gridding may compromise the inherent directional advantages of radial sampling and projection reconstruction, which help preserve spatial resolution in the presence of undersampling [10].

The Polar Fourier Transform (PFT) offers a compelling alternative by computing the 2D FT directly in polar coordinates, thereby eliminating the need for gridding and avoiding interpolation in k-space [13]. In contrast to gridding—which involves multiple corrective steps for non-uniform sampling—PFT provides a streamlined and structurally consistent reconstruction framework. PFT was initially introduced for image reconstruction in computed tomography [14] and later proposed for MRI by Guo and Song [15]. Only after subsequent methodological advancements did PFT become applicable to the reconstruction of radially acquired MRI data [16–18], though its practical deployment and performance in scanner-integrated settings remained largely unexplored.

Preliminary studies suggest that PFT shifts undersampling artifacts toward the periphery of the image, preserving the central region with minimal contamination. This behavior indicates that angular undersampling in radial acquisitions, when combined with PFT, may enable accelerated imaging of small regions of interest. These studies suggest that, beyond a central area of well-preserved resolution, undersampling effects appear as progressive blurring that intensifies with distance from the center, leading to a gradual degradation of spatial resolution in the periphery [16–18]. Consequently, PFT may be especially well suited for MRI applications where the region of interest is substantially smaller than the full field of view, a challenge previously addressed using methods like zoomed EPI with selective excitation [19, 20]. It is also worth examining whether the peripheral manifestation of undersampling artifacts observed with PFT reconstruction relates to insights from prior point spread function (PSF) analyses [6, 7], which—although conducted using gridding—suggest that motion and undersampling artifacts may be displaced from their point of origin by a distance referred to as reduced field of view (rFOV).

Conventional gridding-based reconstructions rely on three corrective steps—k-space interpolation, density compensation, and deapodization—to address the non-uniformity of polar sampling. In the PFT framework, these corrections are not performed as discrete operations; rather, they are either intrinsically incorporated into the structure of the transform or not required at all. This integration streamlines the reconstruction pipeline and may offer practical advantages, particularly under conditions of undersampling.

To deploy PFT effectively in clinical settings, two key factors must be evaluated: (1) its computational efficiency when integrated into a clinical workflow—particularly for inline reconstruction—and (2) the resulting image quality, with emphasis on noise, artifacts, and SNR under conditions of angular undersampling. In this work, we integrate PFT into the reconstruction pipeline of a commercial MRI system and quantify its reconstruction time to assess practical feasibility. Beyond computational performance, we also examine the quality of PFT-reconstructed images in accelerated imaging scenarios—and systematically explore aspects that have been suggested but remain largely underexplored [16, 17]. In particular, we evaluate the spatial dependence of resolution and SNR as a function of acceleration and compare PFT outcomes against the vendor-supplied gridding algorithm, using both phantom and in vivo data to assess image quality and undersampling artifacts.

## Materials and methods

### PFT algorithm and reconstruction framework

The PFT is the polar-coordinate equivalent of the two-dimensional Fourier transform. It consists of two one-dimensional Fourier transforms along the azimuthal direction, connected by a Hankel transform applied along the radial axis, whose kernel is defined by Bessel functions of the first kind. The reconstruction is carried out entirely in polar coordinates and can be summarized as follows [16]:

1 $$f\left(r,\theta \right)={IFFT}_{\theta }\left[{f}_{n}\left(r\right)\right]={IFFT}_{\theta }[{i}^{n}{H}_{n}\left\{{F}_{n}\left(\rho \right)\right\}={IFFT}_{\theta }\left[{i}^{n}{H}_{n}\left\{{FFT}_{\varphi }\left(F\left(\rho ,\varphi \right)\right)\right\}\right].$$

Here, in k-space, ρ denotes the radial distance from the center, and φ is the azimuthal angle. ***F***(ρ, φ) represents the raw k-space data acquired in polar coordinates. In the image domain, ***r*** is the radial distance from the center of the image, and θ is the polar angle. The reconstructed image in polar coordinates is denoted by ***f***(***r***, θ). The symbol *i* is the imaginary unit, and ${H}_{n}$ refers to the Hankel transform of order *n*. (The full derivation of Eq. (1) is available in [16] and not reproduced here.)

Gridding-based reconstructions typically require k-space interpolation, density compensation, and deapodization to address non-uniform sampling and the effects of convolutional interpolation. In contrast, PFT does not involve interpolation in k-space; it avoids the use of a convolution kernel and, consequently, eliminates the need for deapodization to correct for kernel-induced modulation. Of the three corrections, only density compensation remains relevant, and this is intrinsically handled by the Hankel transform, whose mathematical formulation inherently accounts for the radial weighting of samples. This internal handling simplifies the reconstruction pipeline and reduces potential sources of error—especially those arising from interpolation inaccuracies in undersampled data.

Before applying the PFT algorithm, the raw radial k-space data were acquired along each spoke from $-{\mathrm{k}}_{\mathrm{max}}$ to $+{\mathrm{k}}_{\mathrm{max}}$. To conform to the mathematical formulation in Eq. (1), the data were reorganized into a center-out configuration, in which each spoke was split into two halves at *k* = 0. These halves were then used to construct the matrix ***F***(ρ, φ) for φ values that are 180 degrees apart. In case of odd number of samples per spoke, the middle sample *k* = 0, shared by both halves, was duplicated to maintain symmetry. This duplication ensures compatibility with the transform structure and does not introduce any weighting bias in the reconstruction. (A more detailed discussion of this point is provided in the Discussion section.) If the number of samples per spoke is even, the spokes are still split in half and the distance of the middle samples from center of k-space ($\mp \frac{\Delta k}{2}$) is considered in further calculations.

No specific correction for gradient delays was applied in this implementation. While correction techniques developed for gridding-based methods could, in principle, be adapted to PFT, we did not incorporate such corrections to focus on evaluating the intrinsic sensitivity of the PFT reconstruction to gradient-induced imperfections. Each data point in the reorganized k-space dataset is thus uniquely defined by its prescribed radial distance from the center and the angular position of its corresponding spoke. The output of the PFT is an image expressed in polar coordinates.

In the reconstructed image in this framework, the pixel size along the azimuthal (angular) direction increases proportionally with the radial distance from the center. For display and analysis, the reconstructed polar image is interpolated onto a Cartesian grid. Importantly, this coordinate transformation does not enhance actual spatial resolution—choosing a finer Cartesian grid does not yield higher true resolution. Consequently, the apparent pixel size in the transformed image may not accurately reflect the system’s underlying resolution. This limitation is discussed further in the Discussion section.

### Inline implementation

Every MRI platform includes a computational pipeline that reconstructs images based on parameters defined by the acquisition pulse sequence. The computational efficiency of PFT must be sufficient to enable direct integration into this reconstruction pipeline.

**Fig. 1:**
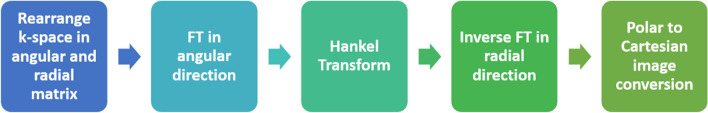
Consecutive steps of the Polar Fourier Transform (PFT) algorithm for reconstruction of radially acquired MRI data. The process includes reorganization of k-space into center-out spokes, a Fourier transform along the azimuthal direction, a Hankel transform along the radial axis, an inverse Fourier transform along the azimuthal direction, and final interpolation from polar to Cartesian coordinates

The main steps of the PFT algorithm (see Fig. 1) were implemented as independent processing modules, replacing the default gridding reconstruction block within the image reconstruction environment. For this study, the PFT algorithm was embedded into the pipeline using the Siemens Image Calculation Environment (ICE) on a C +  + platform. No additional image processing or filtering was applied beyond the final coordinate transformation used to convert the polar image to Cartesian.

After reorganizing the k-space data into center-out spokes (as described earlier), the one-dimensional Fourier transforms were executed using the built-in FFT and inverse FFT functions provided by the ICE libraries.

The other core step—the Hankel transform, which is applied between the two one-dimensional Fourier transforms—requires a kernel based on Bessel functions of the first kind. In the overall workflow of the PFT algorithm, this Hankel step represents the main computational bottleneck, due to the need to evaluate Bessel functions of integer order. Although a series-based definition for Bessel functions exists and is mathematically valid for all input values, its computational cost becomes excessive for large arguments, rendering it impractical for this implementation.

Instead, we implemented a recursive numerical algorithm for evaluating integer-order Bessel functions, as described in [21]. This method operates in two stages. First, Bessel functions of orders zero and one are computed using their asymptotic approximations; then, higher-order Bessel functions are recursively computed from known recurrence relationships with adjacent orders [21].

In our PFT implementation, where the 1D Fourier transform is performed using the FFT, the order of the Hankel transform is directly determined by the number of angular samples (radial spokes) specified in the acquisition protocol [17], and this order remains fixed for a given scan. As a result, the corresponding Bessel function values were computed once per scan using the recursive method and reused across all reconstructions for that scan, including those involving multiple coil channels or temporal repetitions. To further improve computational efficiency, parallel processing was employed, with the Hankel transform for each coil channel executed concurrently on separate CPU threads.

To increase azimuthal sampling density in the final polar image, zero padding was applied along the angular frequency axis prior to the inverse FFT, resulting in interpolation along the angular dimension. For display, the polar image was resampled onto a Cartesian grid using nearest-neighbor substitution—a simple method that avoids interpolation-related smoothing artifacts.

### Data acquisition and image reconstruction

This study was conducted on a 3 T Siemens Prisma scanner, equipped with a 20-channel head/neck coil. Image reconstruction was performed using both the implemented PFT and standard gridding algorithms, each integrated into the MRI system running Syngo MR software version VE11E. In the PFT implementation, Bessel function values were computed during the reconstruction of the first slice of each scan and cached in memory for reuse in subsequent slices, thereby eliminating redundant computations.

To evaluate noise characteristics, artifact behavior, and image contrast, phantom experiments were conducted using both a homogeneous water phantom and the structured ACR phantom. Data were acquired using a 2D radial spoiled gradient echo (GRE) sequence, with the following fixed parameters: pixel size = 0.9 × 0.9 mm^2^, base resolution = 256, and bandwidth = 399 Hz/pixel. Other acquisition parameters were varied depending on the experiment, as summarized in Table 1.

**Table 1:** imaging parameters for data acquisition

Subject	Sequence	Number of spokes	Number of slices	Repetition	FA (degree)	TR (ms)	TE (ms)	Slice thickness
Water phantom	Spoiled GRE	31/63/127/255	3	30	12	15	3	3
Structured phantom (ACR)	Spoiled GRE	31/63/127/255/256 Odd / and even	3	1	12	15	3	3
ACR phantom	SSFP	255 (to compare with human)	3	1	20	4.30	2.14	1
Human	SSFP	63/127/255	20	1	60	4.30	2.14	3

For noise quantification, the water phantom was imaged with 30 repeated acquisitions to enable estimation of the signal-to-noise ratio (SNR) under different undersampling conditions: number of spokes (N_s_) = 31, 63, 127, and 255. The same number of spokes was used for the structured phantom, which was scanned to enable qualitative assessment and evaluation of undersampling-induced streaking artifacts [22].

Gradient imperfections—such as gradient non-linearity and eddy current-induced gradient delays—are known to degrade image quality in radial acquisitions [4, 23]. Therefore, evaluating the sensitivity of PFT to these effects is a critical consideration. Prior studies have shown that the readout direction and spoke ordering can significantly influence the severity of gradient-induced artifacts [23]. Specifically, alternating the readout direction for consecutive spokes has been shown to mitigate these artifacts.

To assess the sensitivity of the PFT reconstruction to gradient delay artifacts, we acquired data using two distinct acquisition schemes: monopolar and bipolar (alternating) readout directions. As a practical simplification, we implemented the monopolar scheme using an even number of spokes uniformly spanning the angular range from 0 to π. For the bipolar configuration, we used an odd number of spokes evenly distributed over the full 0–2π range. This naturally introduces alternating readout directions without requiring explicit reversal in the pulse sequence. The even/odd approach thus allowed us to toggle between acquisition schemes with minimal changes to the sequence. For comparison, the structured phantom was scanned using N_s_ = 255 and 256, with all other parameters held constant between the two schemes.

To evaluate in vivo applicability of PFT, one healthy volunteer underwent brain imaging after providing informed written consent. The field of view (FOV) was set to 23.0 cm, sufficient to capture the entire brain across 20 slices. The in-plane spatial resolution was maintained at 0.9 × 0.9 mm2, consistent with the phantom protocols. A balanced steady-state free precession (bSSFP) sequence was used to generate strong anatomical contrast and to enhance the visibility of potential artifacts.

All phantom and in vivo datasets were reconstructed using the inline PFT pipeline, with acquisition parameters listed in Table 1. Reconstruction times for both PFT and standard gridding were recorded for comparative evaluation.

### SNR and CNR measurement

The spatial noise distribution in images reconstructed using PFT has been reported to be non-uniform [16, 17]. To experimentally characterize this distribution, we used temporal signal variations as a proxy for local noise estimates, assuming that temporal and spatial statistics are equivalent (i.e., ergodic). Accordingly, noise and signal-to-noise ratio (SNR) were estimated on a pixel-wise basis by analyzing the time-series of each pixel from repeated measurements [24, 25]. This approach avoids relying on background regions for noise estimation, which may not be representative in reconstructions like PFT where noise characteristics vary across the image.

Specifically, 30 identical acquisitions were performed on a water phantom, and the SNR at each pixel was calculated using the following equation:

2 $$SNR=\frac{\mu }{\sigma }=\frac{{\sum }_{i=1}^{N}{x}_{i}/N}{\sqrt{{\sum }_{i=1}^{N}({x}_{i}-\mu {)}^{2}/N}}.$$

In Eq. (2), ${x}_{i}$ denotes the pixel intensity at time point *i*, while μ and σ represent the temporal mean and standard deviation of that pixel’s intensity across the time-series. The variable *N* denotes the number of repeated measurements, which was set to 30 in this study. To obtain the SNR for a region of interest (ROI), the pixel-wise SNR values within that region were averaged.

To evaluate image contrast, we calculated the contrast-to-noise ratio (CNR) for both phantom and human datasets. The CNR between two regions *A* and *B* was computed using the following definition:

3 $$CNR= \frac{{S}_{A}-{S}_{B}}{\sigma }=\frac{{S}_{A}-{S}_{B}}{\sqrt{{\sum }_{i=1}^{N}({x}_{i}-\mu {)}^{2}/N}}.$$

Here, ${S}_{A}$ and ${S}_{B}$ represent the average signal intensities of the two regions of interest, and $\sigma$ refers to the noise estimate derived as in Eq. (2).

## Results

### Phantom study

**Fig. 2:**
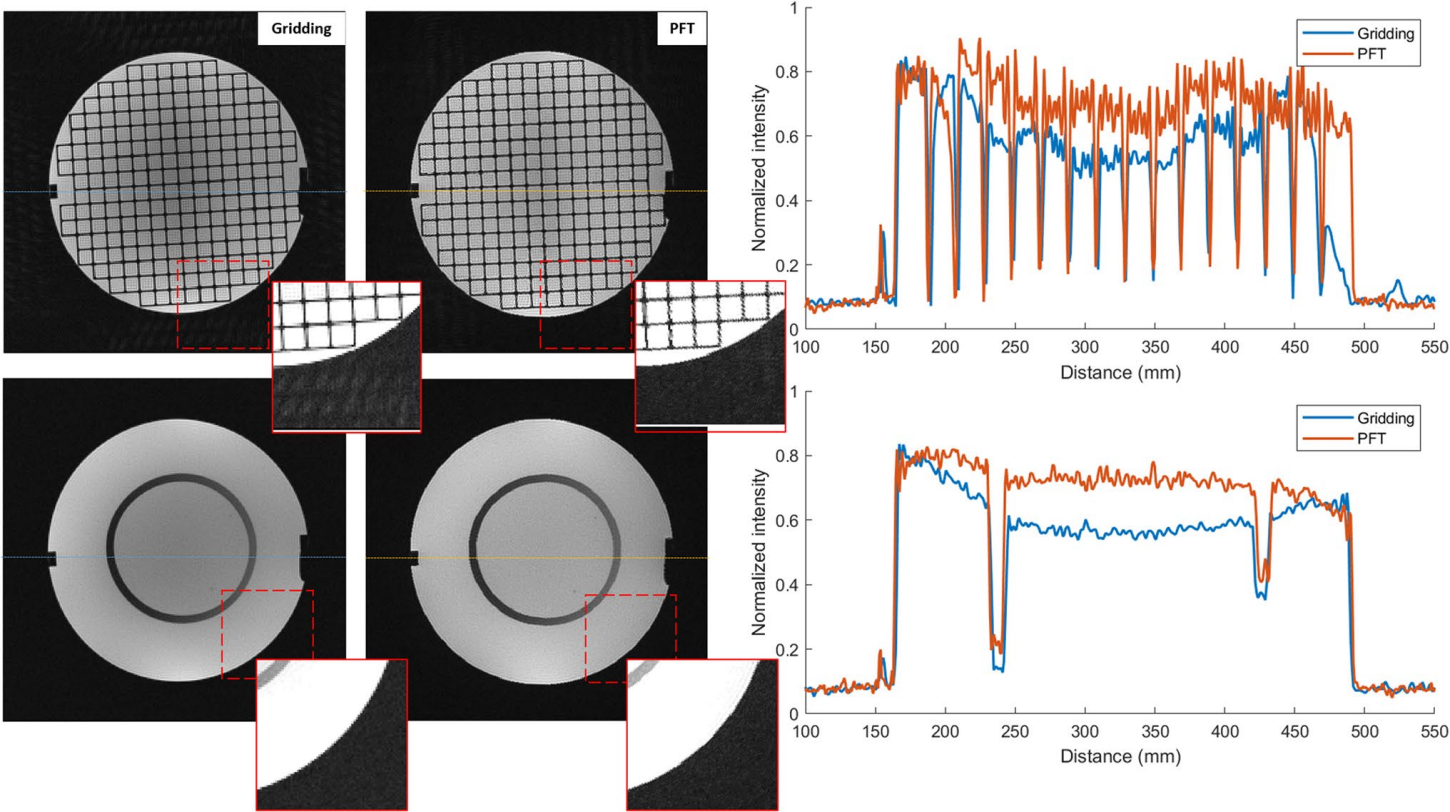
(Left panel) comparison between gridding (first column) and PFT (second column) reconstruction methods is demonstrated for two slices of the ACR phantom. The red dashed squares at the corner of each image show a different pattern of artifacts in the background area. The brightness of the cropped areas is elevated by 40% for better visualization. (Right panel) normalized brightness over one line in each of the reconstructed images (shown by dashed lines) has been plotted to compare the two methods in terms of contrast and signal uniformity

As a demonstration of image quality, phantom images acquired with N_s_ = 255 spokes are shown in Fig. 2. The left panel displays images of a structured phantom from two different slices. For each slice, the same raw dataset was reconstructed by the scanner using both gridding (first column) and PFT (second column) methods. Radial acquisitions are typically performed with an oversampling factor of 2 along the radial direction to prevent aliasing artifacts [26]. For visual consistency, the large circular FOV in the PFT reconstruction was cropped to a square format to match the standard appearance of gridding-based images.

Although the two reconstructed images appear visually similar overall, their background artifact patterns differ noticeably in the magnified views shown in Fig. 2. To facilitate a more detailed comparison, intensity profiles along a representative line from each image are plotted in the right panel of Fig. 2. These results indicate that while both methods preserve structural features, PFT demonstrates improved signal uniformity within the homogeneous water-filled region. In the gridding reconstruction, a mild central intensity dip is observed, which may be influenced by phantom geometry and its effect on low-frequency k-space content. The factors contributing to this difference are discussed further in the Discussion section.

**Fig. 3:**
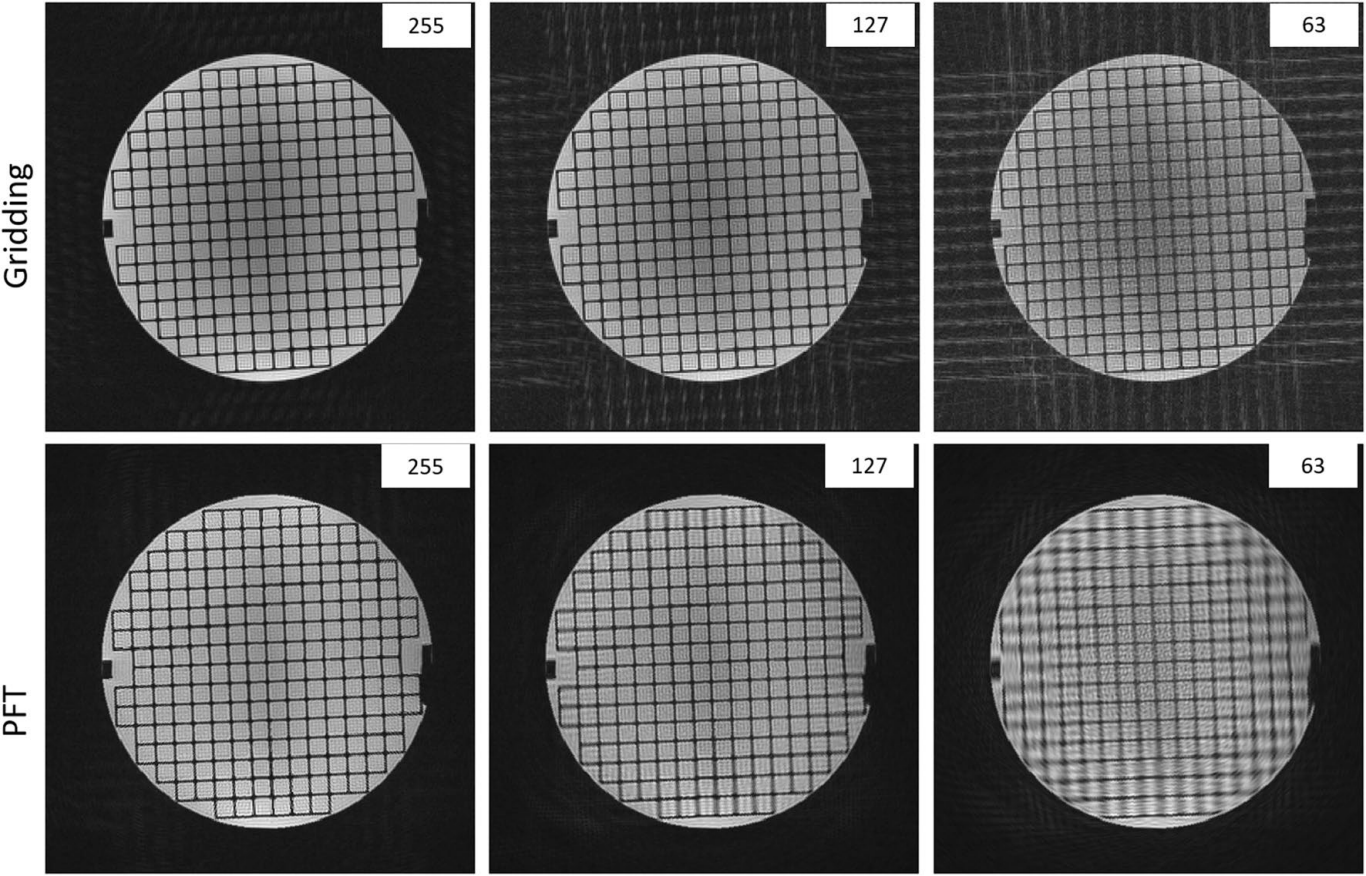
The effect of undersampling on the gridding and PFT reconstructions of radially acquired data. The grid phantom has been imaged with N_s_ = 255, 127 and 63 spokes (left to right). It shows that the effect of undersampling appeared mostly in the form of structured artifacts in gridding. For PFT, however, it results in a blurring in the periphery. Images were normalized regards their total energy

Figure 3 illustrates the impact of undersampling using N_s_ = 255, 127, and 63 spokes, highlighting how the reconstruction method influences artifact behavior in structured phantom images. As N_s_ decreases, clear differences emerge between PFT and gridding. In gridding reconstructions, reducing N_s_ leads to stronger streaking artifacts, particularly in the background, and a progressive decline in image contrast. PFT, by comparison, exhibits a more spatially localized degradation pattern: image blurring becomes increasingly pronounced toward the periphery at lower spoke counts, consistent with its spatially varying point spread function (PSF). Some degree of central blurring is also observed, especially at lower spoke counts, possibly due to contrast loss from signal leakage. This effect is comparable in magnitude to that seen in gridding-based reconstructions at similar level of undersampling. In PFT, peripheral degradation—manifesting as blurring artifacts—remains the most distinctive feature of its undersampling behavior.

**Fig. 4:**
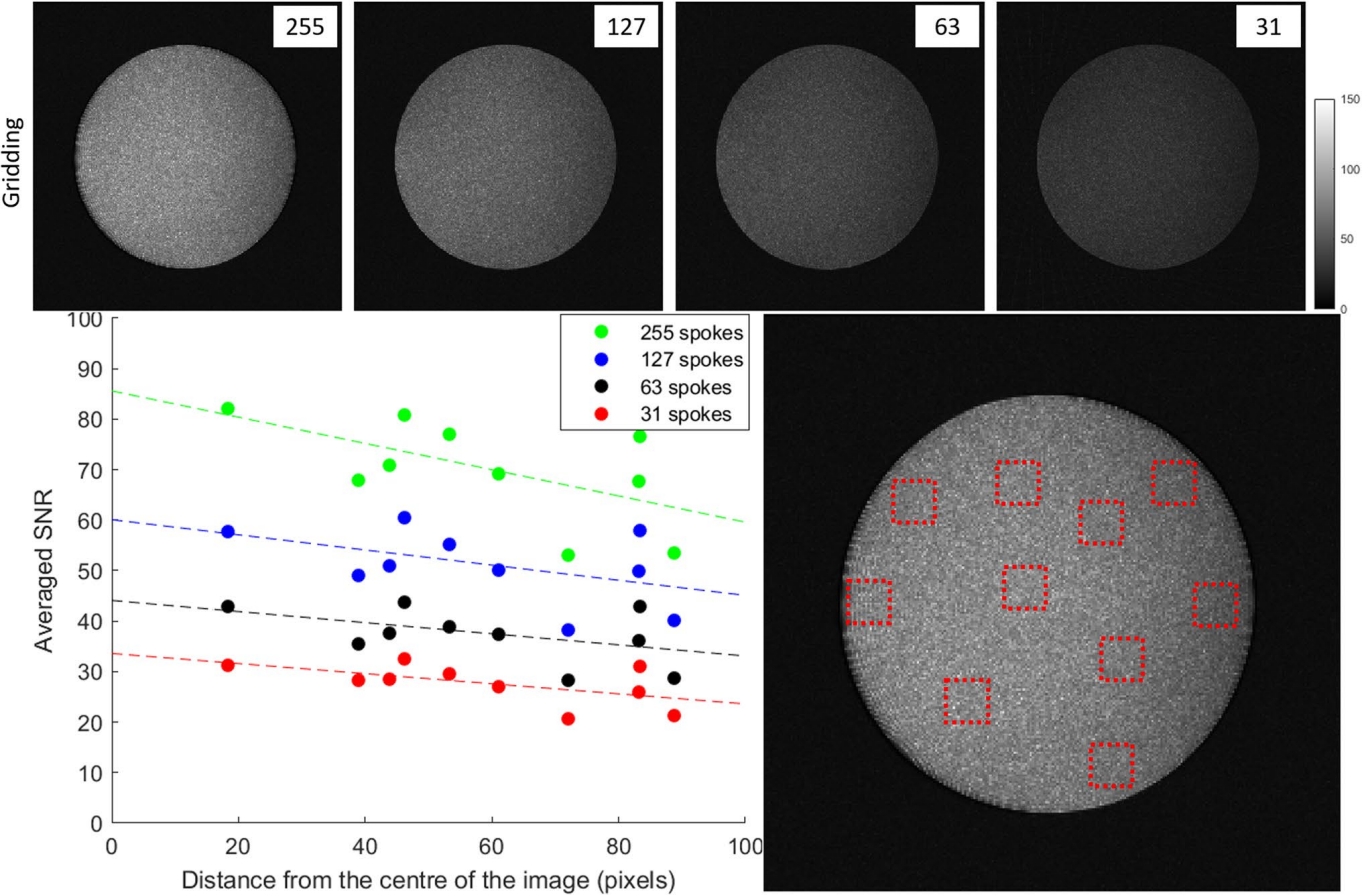
(Top) signal-to-noise ratio maps for images acquired with N_s_ = 255, 127, 63, and 31 from left to right and reconstructed by gridding. (Bottom) the average SNR values of small areas randomly chosen inside the phantom (as shown in the right panel), plotted as a function of their distance from the center of the image. The fluctuation pattern does not show any direct relation between the distance and calculated SNR, suggesting that the changes are mainly caused by coil sensitivities. The pattern is repeated but with some attenuation of SNR for lower number of spokes

To quantitatively assess the SNR behavior in PFT images, we estimated pixel-wise SNR values for a simple water phantom using Eq. (2), as described in the Methods section. The resulting SNR maps for gridding-based reconstructions under different N_s_ values are presented in Fig. 4. Additionally, SNR values were averaged over several predefined regions and plotted versus radial distance from the image center. The right panel of Fig. 4 includes regression lines, indicating that SNR in gridding images shows no appreciable dependence on radial distance, with regression slopes under 3% per centimeter. However, a significant global SNR reduction is evident as N_s_ decreases. This SNR variation shows no consistent dependence on radial distance, suggesting that observed fluctuations are primarily due to coil sensitivity variation rather than spatial properties of the reconstruction. This contrasts with PFT, where the SNR behavior is inherently spatially dependent, as elaborated in the Discussion section.

**Fig. 5:**
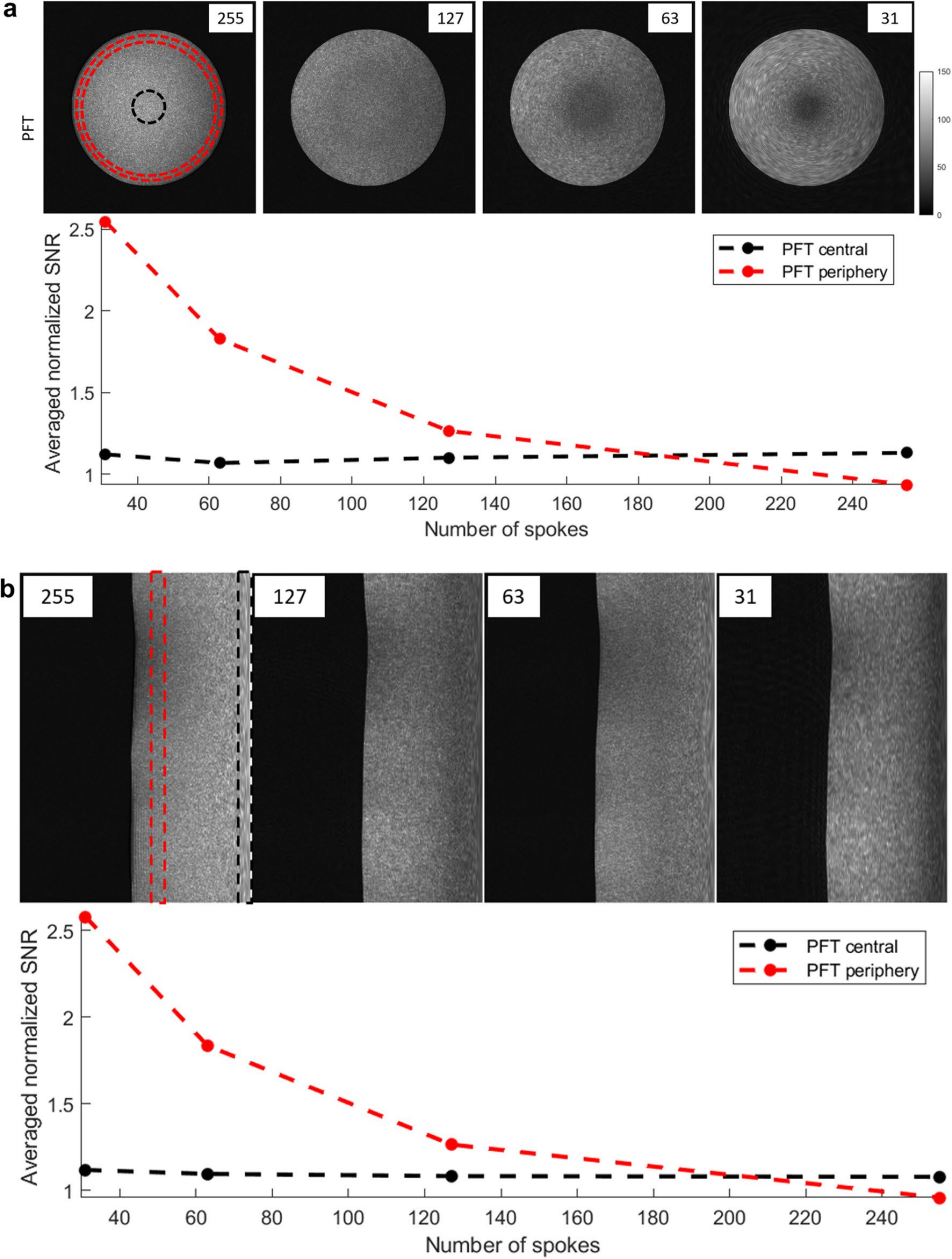
** a** (Top) signal-to-noise ratio (SNR) maps from images acquired with N_s_ = 255, 127, 63, and 31 spokes (left to right), reconstructed using PFT. (Bottom) average SNR values for the central region (within the black dotted ring) and peripheral region (between the red dotted circles) are divided by the corresponding SNR values from gridding reconstructions to highlight the relative performance. In the central region, PFT consistently yields ~ 12% higher SNR than gridding. In peripheral regions, PFT maintains higher relative SNR at lower spoke counts, while gridding exhibits the expected SNR reduction with undersampling. **b** (Top) SNR maps from the same datasets reconstructed by PFT in polar coordinates. Peripheral areas exhibit spatial variability, with certain angular sectors showing reduced SNR. (Bottom) average SNR values in the central (black box) and peripheral (red box) polar regions, divided by corresponding values from gridding images, confirm that the SNR trends observed in Cartesian reconstructions are preserved in the native polar domain

Figure 5 compares SNR behavior in PFT and gridding reconstructions. To eliminate the effects of coil-related non-uniformities, the SNR values from PFT images were divided by the corresponding values from gridding reconstruction, yielding a relative SNR ratio. This comparison shows that relative SNR of PFT, measured with respect to gridding, remains constant in the image center across all spoke counts, while in peripheral regions, it increases with decreasing N_s_—reflecting a trade-off with reduced spatial resolution.

To assess whether image domain interpolation affected the results shown in panel (a), we repeated the SNR analysis using the polar-domain images directly, as shown in panel (b) of Fig. 5. The relative SNR distributions before and after Cartesian conversion (using nearest-neighbor interpolation) remained largely unchanged, indicating that interpolation did not significantly influence SNR trends.

In the image center, the absolute SNR increased proportionally with the square root of N_s_ for both reconstruction methods, consistent with theoretical expectations. Notably, PFT images exhibited approximately 12% higher central SNR than gridding images in this phantom study. In the peripheral regions, gridding images continued to follow the square-root dependence, but SNR in PFT reconstructions remained largely constant, showing no visible dependence on N_s_.

### Human study: anatomical imaging

**Fig. 6:**
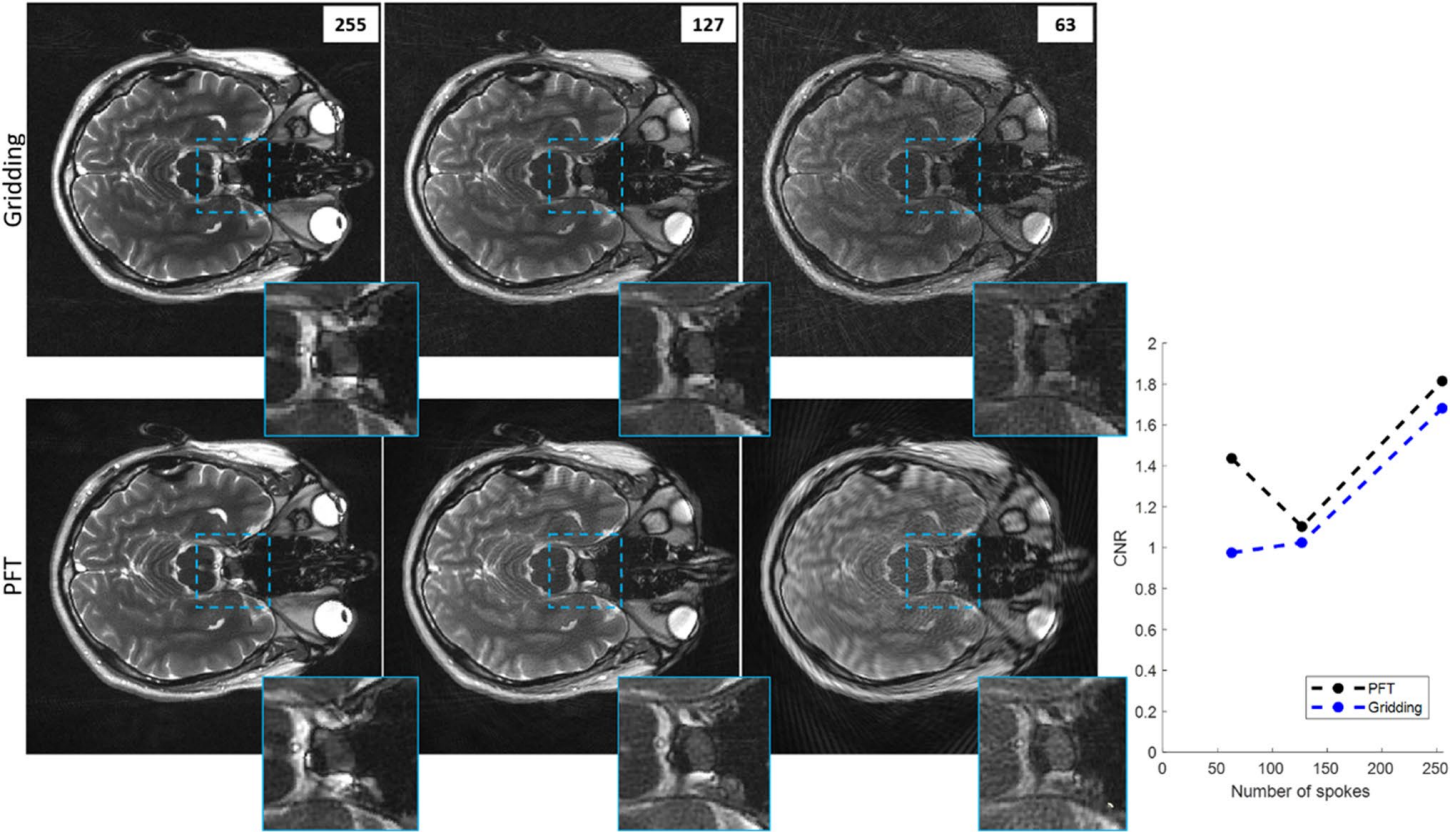
(Left panel) Brain image from a selected slice that contained the pituitary gland (shown with a dashed blue square), for 255, 127, and 63 spokes reconstructed by Gridding (top) and PFT (bottom) methods. The dashed blue square was magnified to better show the image quality at the central part. Even the tiny basilary vein (pointed to by the yellow arrow) is still visible at PFT reconstruction with 63 spokes (better than Gridding with 127 spokes). Images were normalized regarding their total energy. (Right panel) CNR for the pituitary gland plotted for each reconstruction method with respect to different number of spokes. The contrast was specifically calculated for this little gland compared to neighboring nose cavity. The CNR is similar for two methods at higher sampling rates but for N_s_ = 63 increases for PFT based on the fact that the high-resolution area has become so small that it no longer covers the nose cavity. Therefore, the noise inside the cavity drops as the blurring increases and, therefore, CNR increases

The performance of the implemented method was evaluated in vivo on a human brain, as described in the Methods section. For spoke counts of N_s_ = 255, 127, and 63, the corresponding acquisition times were 1.1, 0.55, and 0.28 s per slice, respectively. The PFT reconstruction times per slice under these sampling conditions were measured as 7.39, 3.92, and 2.29 s, respectively. Results for this anatomical imaging study are presented in Fig. 6, which shows a representative slice from the human brain.

At full sampling (N_s_ = 255), both reconstruction methods produced similar visual representations of fine anatomical structures. However, as the number of spokes was reduced, qualitative differences became apparent. In particular, PFT reconstructions exhibited spatially varying image quality, with central regions better preserving anatomical detail when subjected to undersampling. This behavior is advantageous for imaging small targets within a large field of view—such as the pituitary gland in whole-brain scans.

The representative slice in Fig. 6 includes the pituitary gland, which—though located slightly off-center—remains clearly visualized even at N_s_ = 63 using PFT. Notably, the basilar artery, located posterior to the gland, is visible in the PFT-reconstructed images but is not clearly discernible in the corresponding gridding reconstructions—even at higher spoke counts.

The right panel of Fig. 6 displays the contrast-to-noise ratio (CNR), computed using Eq. (3), for the pituitary gland relative to the adjacent nasal cavity. The CNR achieved by PFT reconstruction at 4 × acceleration (N_s_ = 63) is significantly higher than that from gridding. For this analysis, noise was estimated within the nasal cavity. (Notably, estimating noise from outside the skull would result in an even higher CNR for PFT, due to the spatial distribution of noise power—which appears artificially lower in peripheral regions as a result of blurring in PFT, as demonstrated in Figs. 5 and 6).

### Efficiency of algorithm

The Hankel transform constitutes the most computationally intensive component of the PFT algorithm. To mitigate this limitation, runtime was substantially reduced by implementing optimized numerical techniques for computing the Bessel functions required for the Hankel transform. The Bessel function values were computed during reconstruction of the first image and stored as a lookup table in memory. This table, generated only once per reconstruction session, was then reused for all subsequent images acquired under the same protocol— similar to how gridding weights are handled. By excluding Bessel table generation from the main reconstruction loop, computational cost was significantly reduced—particularly for dynamic imaging protocols or multi-slice datasets, which typically maintain fixed acquisition parameters.

PFT-based online reconstruction was performed using the vendor's reconstruction platform on an Intel® Xeon® CPU E5-2620 v4 (2.10 GHz, 16 cores, 1 thread per core, 64 GB RAM). For human brain imaging with 255 radial spokes and 256 samples per spoke, the Hankel transform required 6.1 s, and the total PFT reconstruction time per image was 7.39 s. This performance was made possible through parallelization of the Hankel transform across the receiver (Rx) coil channels.

Consequently, total image reconstruction time was approximately 6.7 × the acquisition duration, underscoring the feasibility of using PFT in near real-time or inline clinical imaging applications.

## Discussion

While the PFT reconstruction algorithm has been formulated and applied offline in recent years, its direct implementation on MRI scanners had not previously been reported, limiting prior validation to simulations or offline tests. In this work, we implemented it directly on the scanner, enabling experimental validation of several previously hypothesized properties of PFT—including central focusing behavior, spatially dependent artifact patterns, and distinct SNR characteristics [16].

We first demonstrated the feasibility of implementing PFT on MRI scanners by achieving practical reconstruction times and adequate computational efficiency. Building on both phantom and human studies, we then systematically compared PFT with gridding reconstruction in terms of artifact behavior and spatial distribution of SNR.

### PSF and spatial dependency in PFT

**Fig. 7:**
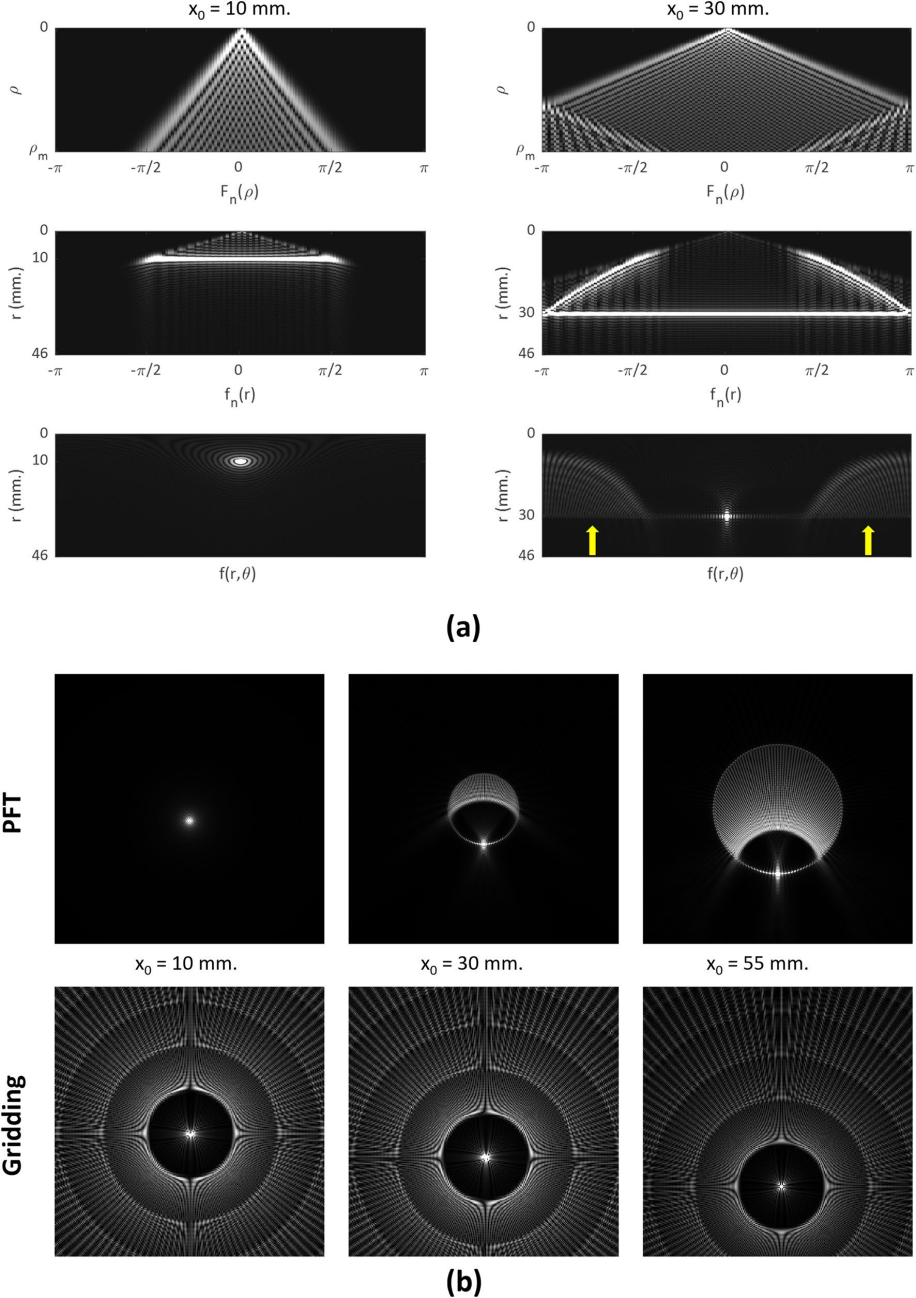
Comparison of the point spread function between PFT and gridding techniques for points located at x_0_ = 10 mm, x_0_ = 30 mm and x_0_ = 55 mm (from left to right respectively). (a) PFT reconstruction in different stages of the algorithm for two different source points located at locations (r,θ) = (10,−π/2) and (r,θ) = (30,−π/2). (b) PFT (top) and gridding (bottom) reconstructions for three different source point located at locations (r,θ) = (10,−π/2), (r,θ) = (30,−π/2), and (r,θ) = (55,−π/2)

To understand the mechanisms underlying the spatially varying behavior observed in PFT reconstructions—including artifact distribution and SNR patterns—we analyzed the system’s point spread function (PSF) using targeted simulations. Specifically, we reconstructed point sources (delta functions) placed at various radial distances from the image center (Fig. 7) to visualize how the reconstruction algorithm responds locally in undersampling conditions. For this analysis, 63 diagonal spokes were used to highlight undersampling effects, with all other acquisition parameters matching those of our experimental studies.

Both PFT and gridding reconstructions were simulated for delta functions positioned at two representative locations—10 mm and 30 mm from the center—corresponding to “central” and “peripheral” regions, respectively. Panel (a) of Fig. 7 shows the results of the PFT reconstruction at each step, illustrating how aliasing artifacts manifest in this method. At this level of undersampling, the peripheral delta function (30 mm) exhibits substantial broadening and aliasing artifacts that are absent near the center (10 mm). These artifacts appear as secondary intensity peaks located symmetrically opposite the original delta location (indicated by yellow arrows).

To facilitate side-by-side comparison, PSFs from three radial distances were also mapped onto a Cartesian grid and shown in panel (b), alongside those from gridding-based reconstructions. The gridding method exhibits a space-invariant PSF characterized by a central circular artifact-free region, known as rFOV, whose radius is calculated as 47.6 mm based on our simulation parameters [7]. In contrast, the PFT reconstruction begins to show aliasing effects beyond a radius of approximately 23.8 mm—corresponding to half the rFOV calculated in gridding.

This comparison highlights a key distinction: gridding reconstructions exhibit shift-invariance across the field of view, enabling image formation to be modeled using convolution with a fixed PSF kernel. Our PFT implementation, however, exhibits strong spatial variation in its PSF—particularly in the presence of undersampling. This space dependency violates the assumptions required for convolution-based modeling with a spatially invariant kernel and calls for spatially adaptive interpretation of the reconstruction behavior.

As the radial distance increases in PFT-reconstructed images, two principal effects emerge:

1. Azimuthal resolution degrades, as reflected in broader PSF profiles.
2. Signal leakage becomes more pronounced, with energy spreading across a wider region, particularly in directions opposite the original point source.

These effects directly influence image resolution as well as the nature and visibility of aliasing artifacts. In gridding, the space-invariant PSF produces structured artifacts—sharp, high-frequency streaks that maintain fixed positions and orientations, regardless of the signal’s origin. This invariance may cause such artifacts to consistently overlap with anatomical features (e.g., vessels or tissue boundaries), amplifying their visibility and making them more visually disruptive. Because of this spatial invariance, prior studies have shown that an object smaller than half the rFOV can be imaged without aliasing [7].

In contrast, PFT exhibits a spatially varying PSF that not only spreads aliasing energy more diffusely but also reduces azimuthal resolution in the periphery. This results in broader, low-frequency artifacts whose shape and orientation vary with location. Owing to this spatial variability, residual aliasing in PFT often appears as subtle intensity variations or noise-like texture, and less like coherent streaks—a behavior reminiscent of the diffuse artifact suppression observed with variable-density sampling [12]. While some structured components may still emerge, the combination of spatial variance and peripheral blurring prevents consistent alignment with anatomical features, thereby reducing the visual salience of the artifacts. As a result, PFT artifacts blend more subtly into the background and, regardless of object size, fine central features—such as the basilar artery in Fig. 6—can remain visible when they fall within the high-resolution central region.

This PSF behavior also helps explain the SNR patterns observed in Fig. 5. Within the central region, azimuthal resolution remains relatively high, preserving fine structural detail. In contrast, moving outward, progressive blurring along the angular direction smooths local intensity fluctuations, resulting in artificially elevated SNR in the periphery. The boundary of this high-resolution region corresponds to rFOV/2 and gradually shrinks with increasing undersampling. The distinct undersampling-induced blurring of PFT—illustrated in Fig. 3—also stems from its spatially varying PSF.

From a complementary perspective, the radial variation in resolution observed in PFT can also be explained as a direct consequence of the polar sampling geometry and the transform structure. In particular, the 1D FFT applied along the azimuthal direction preserves the number of angular samples during the transition from k-space to image space. As a result, pixel size in the angular direction increases proportionally with radius, leading to reduced azimuthal resolution toward the periphery. However, this does not imply enhanced resolution near the center. Despite smaller pixel sizes in central regions, the true spatial resolution is fundamentally constrained by the highest acquired spatial frequency ${K}_{max}$. These smaller pixels reflect interpolation rather than actual resolvable detail.

Maintaining consistent resolution across the entire field of view, as defined by ${K}_{max}$, requires a fully sampled radial acquisition. Based on the Nyquist criterion, this corresponds to ${N}_{spokes}={N}_{Base resolution}*\frac{\pi }{2}$ (≅ 400 in our experiments), which is approximately 56% more than the number of phase-encoding steps used in a Cartesian acquisition with the same base resolution. (Notably, the Nyquist condition for azimuthal sampling corresponds to a pixel size at full FOV equal to 1/${K}_{max}$.) In practice, however, radial imaging is typically performed with a number of spokes no greater than that of a fully sampled Cartesian acquisition, leveraging its robustness to undersampling. To accelerate acquisition further, some degree of artifact is typically accepted. While gridding distributes these artifacts as sharp streaks across the entire image, PFT manifests them primarily as a combination of low-frequency intensity variations, peripheral blurring, and occasional structured (streak-like) artifacts, depending on the interaction between image structure and PSF.

### Dynamic imaging and ROI-focused applications of PFT

The spatially variant behavior of PFT, as established above, offers a distinct advantage for dynamic MRI applications where temporal resolution is critical and the diagnostic region of interest (ROI) is spatially localized. Our results demonstrate that reducing the number of radial spokes in PFT has less adverse impact on image quality within the central region—typically encompassing the ROI—while the periphery undergoes progressive blurring. This leads to a natural “ROI-focusing” effect: image fidelity is preferentially preserved in the center of the field of view, where the region of interest is most often located.

This characteristic makes PFT particularly appealing for dynamic studies, such as perfusion imaging, functional MRI, or contrast-agent tracking, where the ROI is confined to a central organ section or small anatomical region. Peripheral degradation, when it occurs, can often be tolerated or selectively mitigated using techniques such as view-sharing [27] or deep learning-based resolution enhancement. In such cases, any compromise in temporal resolution is localized to the periphery, while the central region retains high temporal fidelity. Moreover, dynamic imaging typically involves reconstructing a series of frames, allowing the one-time computation of Bessel coefficients to be amortized across the entire dataset—further improving computational efficiency.

Importantly, the high-resolution center in PFT does not need to coincide with the scanner isocenter, as the imaging frame can be positioned freely during scan planning to ensure that the ROI is centered within the acquisition field.

### Signal and SNR behavior

The signal intensity profiles in Fig. 2, along with the uniform brightness of the water region in Fig. 3 and the higher measured values in Fig. 5, consistently show that PFT reconstruction yields stronger signal intensity in the central image region compared to gridding. This enhancement aligns with the concentration of low-frequency content near the image center—where true signal often dominates—and appears consistently across different undersampling levels and experimental conditions.

One plausible contributor to this trend is the treatment of low-frequency information. Gridding reconstructions typically apply density compensation to correct for the non-uniform k-space sampling inherent in radial acquisitions. This step is often implemented as a pre-weighting of the k-space data that functions similarly to high-pass filtering and may unintentionally attenuate true low-frequency signal. In contrast, PFT performs spatial encoding natively in the polar domain, where sampling density is inherently addressed through the Hankel transform itself. This structure avoids explicit density compensation and may therefore preserve low-frequency content more effectively, potentially leading to enhanced signal retention in regions dominated by low-frequency content.

At the same time, other mechanisms may also contribute to the observed signal behavior, though they reflect side effects rather than intentional design features. One such factor is gradient delay, which, if uncorrected, causes slight shifts in the k-space trajectory and, based on our simulations, may lead to biased signal intensity near the image center. While our gridding pipeline incorporates gradient delay compensation, our current PFT implementation does not. Another contributor may be PSF-related signal leakage: the space-variant point spread function in PFT tends to spread peripheral energy inward in conditions of undersampling. Both mechanisms could artificially boost central signal levels, not by improving reconstruction fidelity but by redistributing peripheral signal content. These effects, therefore, represent potential confounding influences that complicate the interpretation of signal enhancement.

Given these multiple overlapping factors—some inherent to the reconstruction strategy, others introduced by system imperfections or PSF behavior—the current observations should be interpreted as empirical findings rather than definitive conclusions. While the evidence points toward meaningful SNR advantages for PFT in central regions, further analytical work is needed to fully characterize these effects and disentangle their origins. Such studies could guide future refinements to both PFT methodology and its implementation in dynamic or high-precision applications.

### Practical considerations and reconstruction efficiency

The primary computational cost in PFT reconstruction stems from the Hankel transform. In our implementation, runtime was significantly improved by precomputing Bessel function values and reusing them across reconstructions. Nevertheless, high-resolution PFT reconstructions currently require approximately 6–9 × the acquisition time.

This overhead is partially offset by system dead time, such as during positioning or updating acquisition parameters between successive sequences— a window that can be effectively utilized for computation. Further acceleration is feasible through parallel processing or deployment on dedicated hardware platforms, such as system-on-chip (SoC) architectures, which have previously demonstrated 10–100 × speed improvements for image reconstruction tasks [28].

Notably, dynamic protocols benefit even more from these optimizations, as Bessel values only need to be computed once and can be reused across multiple time frames—further enhancing overall reconstruction efficiency.

### Limitations and future work

The Hankel transform remains the primary bottleneck in PFT reconstruction. While our kernel-based acceleration strategy improved performance, alternative methods—such as numerical approximations of the discrete Hankel Transform—may further reduce complexity. Reference [29] introduces a discrete polar Fourier transform using special radial grids based on Bessel function zeros. While mathematically robust, such sampling schemes are incompatible with the uniform radial readouts typically used in MRI.

A more viable solution may lie in custom SoC platforms [28], which could substantially accelerate Bessel and Hankel computations.

**Fig. 8:**
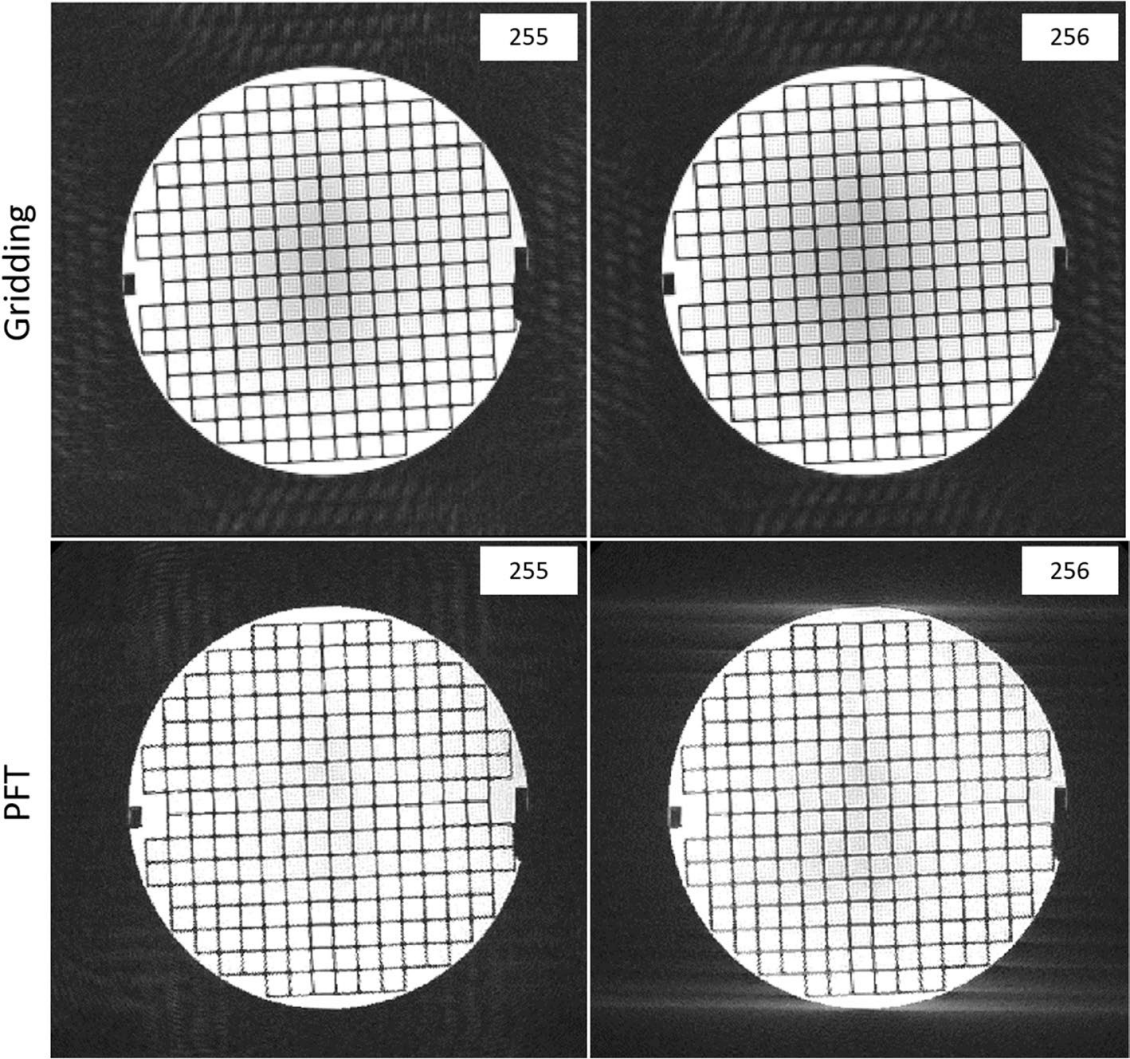
Comparison of acquisitions with odd and even number of spokes in different reconstruction techniques. PFT reconstruction with an even number of spokes shows the gradient delay artifact as two pronounced bright parallel lines on the top and bottom of the phantom edges, while for odd number, the artifact is not as evident. (The contrast and brightness of the whole image are increased by 40% for a better visualization of artifacts.)

A critical challenge in radial imaging is gradient delay, which causes trajectory deviations and artifacts [29–32]. In scanner-based reconstruction (gridding), this issue appears to be addressed through built-in correction algorithms, as evidenced by the absence of characteristic gradient-induced artifact patterns often seen in uncorrected radial data. In contrast, our PFT implementation did not include any gradient delay correction to evaluate its intrinsic sensitivity to such imperfections. In principle, delay correction strategies similar to those used in gridding—such as trajectory calibration or precompensation—could be adapted for PFT, although they were not applied in this study. Figure 8 shows that the associated artifacts, which were clearly visible under monopolar acquisition, were significantly reduced by simply alternating the gradient direction. This straightforward adjustment proved remarkably effective—reducing gradient delay artifacts to a level lower than that observed in gridding-based reconstructions.

Although not a primary focus of this study, the extension of PFT to accommodate non-uniform angular sampling, such as Golden Angle acquisitions, is conceptually straightforward. In prior work [33], where Golden Angle sampling was employed, PFT was tested in parallel and found to be compatible with the proposed reconstruction framework, though the final results were not explicitly reported. Those preliminary tests indicated that even with the standard PFT—without correction for angular aperiodicity—the reconstruction quality remained acceptable. More accurate outcomes could be achieved by adapting the angular transform to handle non-uniform sampling directly, albeit with additional computational complexity.

As a methodological study, this work focuses on artifact behavior, resolution, and SNR/CNR characteristics under undersampling rather than clinical evaluation. Broader studies involving diverse subjects or clinically relevant endpoints remain an important direction for future work.

Finally, estimation errors from Bessel function computation and interpolation should be noted. Our recursive Bessel function evaluation was benchmarked against MATLAB’s built-in routines and showed a maximum absolute error of 1.41 × 10⁻⁶, which is negligible for imaging purposes. Likewise, the polar-to-Cartesian interpolation step introduced minimal artifacts due to the smoothness of the underlying image and the use of zero padding before the final Fourier transform. Nevertheless, more advanced interpolation schemes could improve numerical precision, though possibly at the cost of introducing smoothing effects that could complicate quantitative interpretation.

## Conclusion

Radial trajectories in MR imaging offer unique benefits for accelerated acquisition, particularly when the region of interest (ROI) is smaller than the full FOV. However, to preserve image quality under such conditions, specialized reconstruction methods are required. In this study, we implemented and integrated an algorithm for the PFT reconstruction directly into the scanner’s reconstruction pipeline and demonstrated its feasibility for inline use within clinical workflows. While current reconstruction times remain longer than ideal, these inefficiencies are expected to diminish significantly with continued algorithmic refinement and the adoption of hardware-accelerated platforms.

A key feature of the PFT method is its spatially variant behavior, which becomes especially advantageous when subjected to undersampling. As illustrated through the point spread function (PSF) and confirmed by experimental data, PFT tends to produce more diffuse, less-structured reconstruction artifacts across the image. Even in the central region—where some residual artifacts may still appear—its incoherent and spatially varying nature rarely interferes with visual depiction of key anatomical features, thereby reducing visual interference and preserving image interpretability. This effect is closely tied to the space-dependent resolution characteristics of the PFT framework, where higher azimuthal resolution near the center complements the diagnostic focus of many radial acquisitions.

In the present work, no explicit gradient delay correction was applied. This choice allowed us to evaluate the method’s inherent robustness to such imperfections, and simple alternation of spoke polarity proved sufficient to suppress the resulting artifacts. Likewise, while PFT is compatible with other acceleration strategies, the current implementation focused solely on its ability to exploit the FOV-to-ROI ratio without additional techniques. Even under aggressive undersampling, the method consistently maintained high-quality image features within the small ROI at the center.

While these findings support PFT as a practical and effective reconstruction strategy for radial MRI, several areas remain for future development. These include integration of gradient delay correction, adaptation to non-uniform angular sampling (e.g., Golden Angle), extension to 3D polar acquisitions, as well as further hardware and computational optimizations.

## Data Availability

Upon publication, the codes created during this research will be made available (see GitHub repository: https://github.com/Nasiraei/pft-radial-mri-magma-2025). Access to the Siemens codes should be obtained through the C2P agreement.
